# Approaches, Progress, and Challenges to Hepatitis C Vaccine Development

**DOI:** 10.1053/j.gastro.2018.08.060

**Published:** 2019-01

**Authors:** Justin R. Bailey, Eleanor Barnes, Andrea L. Cox

**Affiliations:** 1 Division of Infectious Diseases, Johns Hopkins University School of Medicine, Baltimore, Maryland; 2 Peter Medawar Building for Pathogen Research, Nuffield Department of Medicine and the Oxford NIHR Biomedical Research Centre, Oxford University, UK

**Keywords:** HCV, Viral Hepatitis, Vaccines, Prophylactic Vaccination, Ad5, adenovirus serotype 5, bNAb, broadly neutralizing antibodies, CD81bs, CD81 receptor binding site, ChAd, chimpanzee adenovirus, DAA, direct-acting antiviral, HCV, hepatitis C virus, HCVcc, HCV derived from cell culture, HCVpp, HCV pseudoparticles, HVR1, hypervariable region 1, mAbs, monoclonal antibodies, MVA, modified vaccinia Ankara, NAbs, neutralizing antibodies, NS, nonstructural, PD-1, programmed cell death 1, PWID, people who inject drugs, VLP, virus-like particle

## Abstract

Risk factors for hepatitis C virus (HCV) infection vary, and there were an estimated 1.75 million new cases worldwide in 2015. The World Health Organization aims for a 90% reduction in new HCV infections by 2030. An HCV vaccine would prevent transmission, regardless of risk factors, and significantly reduce the global burden of HCV-associated disease. Barriers to development include virus diversity, limited models for testing vaccines, and our incomplete understanding of protective immune responses. Although highly effective vaccines could prevent infection altogether, immune responses that increase the rate of HCV clearance and prevent chronic infection may be sufficient to reduce disease burden. Adjuvant envelope or core protein and virus-vectored nonstructural antigen vaccines have been tested in healthy volunteers who are not at risk for HCV infection; viral vectors encoding nonstructural proteins are the only vaccine strategy to be tested in at-risk individuals. Despite development challenges, a prophylactic vaccine is necessary for global control of HCV.

**Figure:**
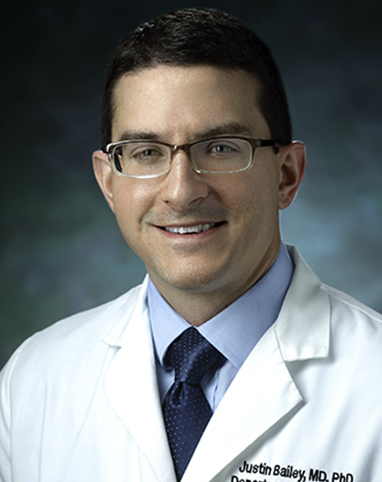
Justin R. Bailey

**Figure:**
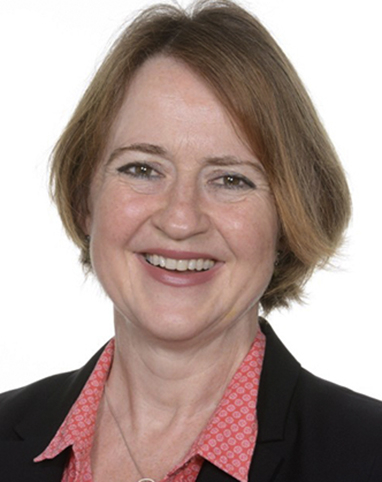
Eleanor Barnes

**Figure:**
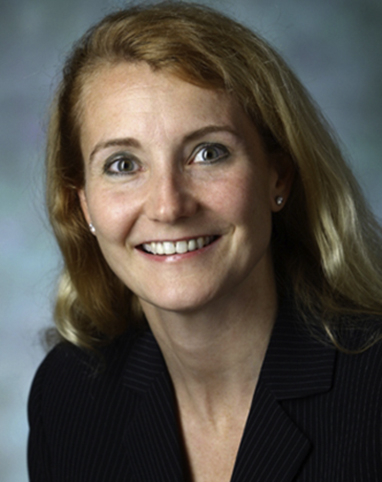
Andrea L. Cox

The advent of all oral, interferon-sparing direct-acting antivirals (DAAs) that cure hepatitis C virus (HCV) infection has transformed treatment, particularly in high-income countries. Although DAAs have fueled optimism for global control, several limitations of treatment make development of a preventive vaccine necessary to achieve that goal. HCV infections are rarely symptomatic before the onset of advanced liver disease, and HCV screening is rare in most parts of the world, so most persons with HCV infection are not identified.^1^ In addition, the cost of and practical aspects to delivering therapy result in only a subset of those diagnosed being treated. HCV treatment has been decreasing globally since its peak in 2015, as the HCV-infected people easiest to access have been treated, leaving those more difficult to access without treatment (John McHutchinson and Diana Brainard, Gilead Sciences, personal communication; 2018).

Some treated individuals have developed resistance to DAAs, and transmission of resistant HCV variants was documented in clinical trials before DAAs were even approved.^2^ With expansion of treatment to patients less able to take medication reliably, antiviral resistance is likely to become more common. Furthermore, liver disease can progress and cancer can develop despite cure of the HCV infection in patients with cirrhosis. So, treatment does not eliminate all of the consequences of HCV infection and prevention of chronic infection offers significant advantages over treatment.

Despite increased cure rates with DAA, HCV elimination continues to be difficult due to reinfection. Immunity after effective treatment has been shown to be insufficient to prevent reinfection with HCV in individuals with ongoing risk of infection, including people who inject drugs (PWID), men having sex with men, and health care workers with frequent exposure to blood and bodily fluids.^3^, ^4^, ^5^, ^6^ Rates of reinfection in these populations vary, but are high when those most at risk of transmitting infection are treated, in part as a means to interrupt transmission. A recent study in PWID treated while actively injecting showed 6-month and 18-month reinfection rates of 12.6 and 17.1 per 100 person-years, respectively.^7^ PWID, men who have sex with men, health care workers, infants born to HCV-infected mothers, and those living in the many countries with high HCV incidence would be expected to benefit from a preventive HCV vaccine. The effects of prophylactic vaccines with varying levels of efficacy and delivery strategies have been modeled.^8^, ^9^, ^10^ Based on these models, high vaccination rates of high-risk seronegative PWID, even with a vaccine with only 30% efficacy, would have significant effects on transmission.

Global control will require annual rates of cure that are consistently and significantly higher than new HCV infection rates. Few countries are on target to eliminate HCV as a public health problem by 2030, the goal set by the World Health Organization in 2016, and nearly 60% of surveyed countries had more infections than cures in 2016.^11^, ^12^ Consequently, control is unlikely to occur without improved focus on and success in reducing the number of new HCV infections in addition to cure. An effective preventive vaccine would have a significant effects on HCV incidence and would provide a major advance toward global HCV control. However, there are barriers to development, including limitations to HCV culture systems, virus diversity, limited models, and at-risk populations for testing vaccines, and incomplete understanding of protective immune responses.

## Feasibility of Traditional Approaches for HCV Vaccine Design

Generation of live-attenuated and inactivated whole virus vaccines has been effective against other viruses, but neither strategy is feasible for generating HCV vaccines. The inability to culture HCV (until recently) and ongoing limitations of HCV culture systems have posed challenges to production of a live-attenuated or inactivated whole HCV vaccine.^13^ Culture strains of HCV have adaptive mutations that increase replication efficiency in vitro with unknown effects on replication in humans. Live-attenuated vaccines against other viruses have been generated in 2 primary ways: by passage of virus in nonhuman primate cell lines in which natural variants can arise; these have reduced replication in human cells, and by genetic deletion or inactivation of virulence factors. However, HCV does not replicate at high levels in nonhuman primate cell lines and virulence factors for HCV have not been defined. Practical production aspects and the risk of causing HCV infection with live-attenuated vaccines limit their utility.

## HCV Genetic Diversity

**Figure 1:**
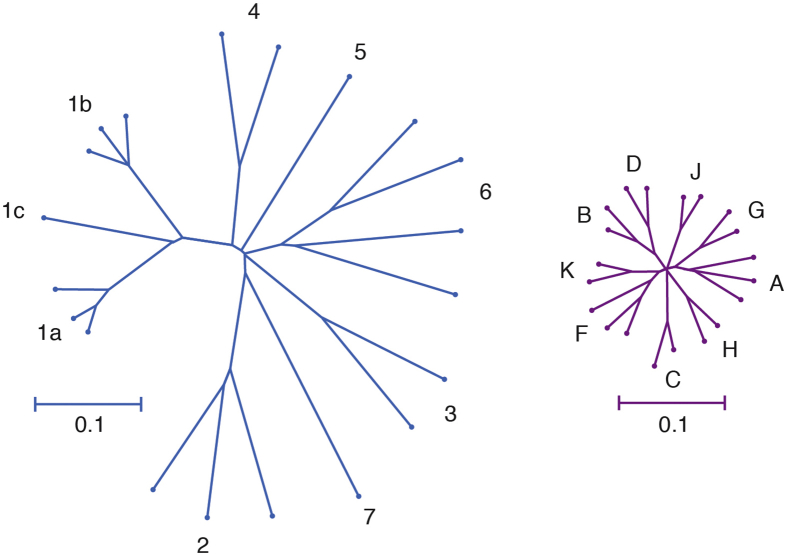
Genetic diversities of HCV and human immunodeficiency virus 1 (HIV-1). Phylogenetic trees of reference full-genome HCV (left) and HIV-1 (right) nucleotide sequences downloaded from the Los Alamos National Laboratory sequence database. Trees were inferred using the Neighbor-Joining method, with branch lengths drawn to scale. Genotypes or subtypes are labeled. Trees are on the same scale. The HCV tree is based on 8940 positions, and the HIV-1 tree is based on 8193 positions. Distances were computed using the Tamura-Nei method. Analyses were performed in MEGA7.^151^

A principal challenge for HCV vaccine development is the extraordinary genetic diversity of the virus. With 7 known genotypes and more than 80 subtypes, the genetic diversity of HCV exceeds that of human immunodeficiency virus-1 (Figure 1). HCV strains from different genotypes differ, on average, at approximately 30% of their amino acids, whereas different subtypes within each genotype differ at an average of approximately 15% of their amino acids.^14^, ^15^, ^16^ In addition to diversity among genotypes and subtypes, immune selection and the error-prone polymerase of the virus generate a diverse quasispecies of related but genetically distinct viral variants within each infected individual, presenting many opportunities for selection of viral variants with resistance to T-cell and antibody responses.^17^, ^18^, ^19^, ^20^, ^21^, ^22^, ^23^ Several recent studies have demonstrated that antibody resistance can arise from mutations either within or distant from antibody binding epitopes, providing the virus with additional mechanisms of immune escape.^24^, ^25^, ^26^ Given this viral diversity within and between infected individuals, vaccine induction of very broadly reactive immune responses or the generation of immune responses that target genetically conserved regions of the viral genome may be required for protection against HCV infection or persistence.

## Challenges to Testing HCV Vaccines

Another barrier to HCV vaccine development is the lack of in vitro systems and immunocompetent small animal models that facilitate determining whether vaccination induces protective immunity.^13^ The discovery of an HCV-like virus, the rat *Hepacivirus*, will generate a new small animal model for vaccine testing. The limitation of this model is that although this virus is structurally analogous to HCV, it has limited sequence homology with HCV.^27^, ^28^ Although the success of DAA therapy has resulted in discussions of HCV challenge studies, in which vaccinated subjects would be intentionally infected with HCV, it is not clear how those infections would be achieved. Primary HCV isolates have limited ability to replicate in tissue culture, restricting their production. In addition, primary HCV isolates are not expressed in good manufacturing process-compliant cell lines and do not represent the viral diversity of the quasispecies circulating in natural infection. Direct infusion of HCV-infected human plasma might be considered, but would require careful screening for other pathogens and, even with thoughtful selection of inoculum levels and HCV genotypes, might fail to completely recapitulate natural exposure.

An effective vaccine is therefore difficult to validate unless it is tested in populations with a predictably high risk for HCV infection. Although HCV transmission occurs through blood transfusion and invasive medical procedures, identifying people who could be at risk of transmission via those routes and vaccinating them before exposure is not feasible. PWID are at predictably high risk with the incidence of HCV infection (ranging from 5% to 25% per year), so they could be a suitable test population for HCV vaccines, and there is a continued need for prevention of HCV infection in this population.^29^ However, few studies have successfully managed the identification, enrollment, and prospective monitoring of PWID before onset of acute HCV infection.^30^, ^31^ These cohorts will likely remain of critical importance to vaccine testing and help us increase our understanding of the host responses required for protective immunity. Knowing what immune responses indicate protective immunity would permit testing of candidate vaccines in healthy adults not at risk for infection first. With correlates of protection known, only those vaccines that elicit effective immune responses would advance to testing in at-risk populations for verification of protection, permitting judicious use of these PWID cohorts. Although our incomplete understanding of protective immunity against HCV is a barrier to vaccine development, studies have provided substantial evidence that protective immunity does exist.

## Evidence for Protective Immunity

Spontaneous clearance of HCV infection occurs in approximately 25% of acutely infected individuals.^32^ Chimpanzees and humans who spontaneously control an initial HCV infection can develop recurrent HCV viremia following additional HCV exposure, so spontaneous clearance of primary HCV infection does not generate sterilizing immunity.^33^, ^34^, ^35^, ^36^, ^37^, ^38^, ^39^, ^40^, ^41^, ^42^, ^43^, ^44^ The discovery that spontaneous immunologic control of HCV does not always result in protective immunity initially decreased confidence that prophylactic vaccination was possible. However, clearance of multiple infections with homologous and heterologous virus has been observed in chimpanzees and humans.^35^, ^40^, ^41^, ^42^, ^44^, ^45^

Furthermore, reinfections are cleared more often than primary infections. Reinfected PWID control secondary HCV infections approximately 80% of the time, essentially the reverse of rates of clearance vs persistence observed in primary infection.^40^ Although genetic traits and other unmodifiable traits affect overall clearance rates, they would not alter the pace of clearance in reinfection. In contrast, adaptive immune responses would be expected to result in more rapid control of reinfection than occurs in the initial infection. Supporting the hypothesis that adaptive immunity induced in control of a primary infection leads to more effective clearance of subsequent infections, Lanford et al^46^ reported decreased duration and magnitude of viremia in chimpanzees in reinfection vs primary infection, regardless of whether the chimpanzees were infected with homologous or heterologous viruses.

HCV reinfection in humans is also characterized by a reduced peak and duration of viremia compared with initial infection of the same person.^40^, ^47^ Reinfection was associated with broadened cellular immune responses compared with primary infection and the presence of broadly cross-reactive neutralizing antibodies (NAbs).^40^

The more rapid and effective control of viral replication with subsequent exposures, compared with the initial exposure, indicates that adaptive immunity is induced and, although not sterilizing, protects against chronic infection. Additional research on effective immune control of HCV is needed to develop a vaccine, including studies of people with repeated spontaneous control of diverse HCV infections over time. Decades of research have revealed that HCV-specific CD4^+^ helper T cells, CD8^+^ cytotoxic T cells, and antibodies all play a role in protection against persistent HCV infection. Vaccine strategies to induce all 3 adaptive immune responses are in development.

## T-Cell–Mediated Protection

Data from human and chimpanzee studies have indicated that HCV-specific CD4^+^ and CD8^+^ T cells are crucial in control of primary and secondary HCV infections. There is indirect evidence for T-cell control from genetic studies, which demonstrated an association between HCV clearance and specific class-I and class-II HLAs, which present HCV peptides to CD8^+^ and CD4^+^ T cells, respectively.^48^, ^49^ A vigorous and multispecific proliferative CD4^+^ T-cell response against HCV proteins has been correlated with spontaneous control of acute HCV infection.^50^, ^51^, ^52^, ^53^ Schulze zur Wiesch et al^50^ demonstrated that broadly directed HCV-specific CD4^+^ T cells are detectable during early stages of infection, regardless of outcome. However, CD4^+^ T cells have functional defects during early stages of chronic HCV infection, and rapidly become undetectable with progression, whereas CD4^+^ T cells are more often maintained with virus clearance. The ineffective CD4^+^ T cells are thought to impair the response of CD8^+^ T cells, and are associated with persistent infection, T-cell exhaustion, and selection of HCV variants with escape mutations in class I epitopes.^53^

Generation of an effective memory response is required for successful vaccination. Reinfection studies provide evidence for the important role of T-cell memory in HCV control. Antibody-mediated depletion of CD4^+^ T cells before reinfection of 2 immune chimpanzees resulted in persistence of HCV despite functional intrahepatic memory CD8^+^ T-cell responses.^54^ Antibody-mediated depletion of CD8^+^ T cells immediately before the third infection of 2 chimpanzees that had previously cleared infection resulted in prolonged viremia, which was controlled only when CD8^+^ T cells were again detectable in the liver.^38^ Similarly, protection against viral persistence in recurrent HCV infection in PWID was associated with broadening of the T-cell response and the expansion of effector memory T cells at the peak of the T-cell response.^40^, ^55^ In addition, early-stage high-level expression of a molecule associated with exhaustion, programmed cell death 1 (PD-1) on HCV-specific CD8^+^ T cells was associated with persistence of reinfection, consistent with findings from studies that associated high expression of PD-1 on T cells during early stages of primary infection with progression to chronic infection.^55^, ^56^, ^57^

Dominant epitope regions of HCV strains isolated from patients with persistent reinfection had sequence variations that were not recognized by preexisting memory T cells. This observation is consistent with escape observed in association with viral persistence following primary infection. Together, these studies demonstrate that memory CD4^+^ and CD8^+^ T cells are required for protective immunity on re-exposure to HCV.

## Trials of Vaccines Designed to Elicit T-Cell Responses

It is a challenge to translate what we have learned about protective T-cell responses to design of vaccines that elicit these responses. The most effective vaccines might induce sterilizing immunity, but most HCV-associated diseases are caused by chronic infection. Immune responses that prevent persistence of infection could decrease HCV morbidity and mortality without reducing incidence of infection. The feasibility of testing vaccines that reduce rates of chronic HCV infection, rather than reducing incidence of infection, has been studied. Reducing viremia during the early acute phase of infection with a vaccine could reduce HCV transmission by lowering the residual infectious virus titers in injecting equipment.^58^, ^59^ So, it might be reasonable to lower the bar for vaccine development, setting goals of decreased persistence or population HCV RNA levels to effect reductions in disease sequelae and transmission.

Another approach is to target the relatively conserved viral proteins within the nonstructural region of the genome to induce a broad T-cell response, with and without including envelope glycoprotein as a target. The nonstructural (NS) proteins (NS3, NS4, and NS5) are more conserved across HCV genotypes than the envelope glycoproteins and are the dominant targets of CD8^+^ T cells.^60^ A variety of strategies have been developed to introduce NS protein antigens in an immunogenic way, including DNA-based immunization, DNA priming followed by recombinant virus vector or HCV protein boosting, recombinant adenovirus priming and DNA boosting, combinations of replicating and nonreplicating recombinant viruses for prime and boost, virus-like particles (VLPs), hepatitis B virus surface antigen–HCV recombinants, and pooled synthetic class I peptide epitopes or peptides incorporated in lysosomes.^29^, ^61^, ^62^, ^63^ An HCV vaccine was recently generated that specifically targets conserved regions of the HCV genome and uses simian adenoviral vectors to generate T cells that target multiple HCV genotypes.^64^ Most candidate vaccines have elicited humoral and cell-mediated immune responses in rodents, and a small subset of candidate vaccines elicit robust CD8^+^ T-cell–mediated immunity in macaques.^65^, ^66^, ^67^, ^68^, ^69^, ^70^, ^71^

Fewer candidate vaccines have been assessed for immunogenicity and ability to protect chimpanzees from HCV. These vaccines include VLP comprising the HCV E1, E2, and core proteins; recombinant nonstructural proteins, formulated with the ISCOMATRIX adjuvant; and genetic vaccines encoding nonstructural proteins.^61^, ^62^, ^72^, ^73^, ^74^ The effects of these vaccines in chimpanzees varied among the studies, which included fewer than 6 vaccinated animals per study.^61^, ^62^, ^72^, ^74^, ^75^ The vaccines encoding envelope glycoproteins induced antibody as well as T-cell responses, but none provided sterilizing immunity.^61^, ^72^ Although not uniformly providing sterilizing immunity, these vaccines reduced primary viremia after challenge with HCV by as much as several orders of magnitude.^61^, ^62^, ^72^, ^73^, ^74^, ^75^ A meta-analysis of all the data from chimpanzee vaccine trials showed that suppression of acute-phase virus replication was associated with recall of vaccine-primed T cells, but the levels of induced T-cell responses did not correlate with vaccine success.^75^ The analysis also noted that vaccines that contained only structural proteins had clearance rates that were significantly higher than vaccines that contained nonstructural components. However, the vectors and antigens used, the timing of vaccination prime and boost, and the timing and identity of challenge HCV viruses varied among the studies, limiting our ability to pool data, compare results from these studies, or conclude that inclusion of envelope proteins is required for a successful vaccine.

Most chimpanzee vaccine studies have shown reduced HCV persistence rates in vaccinated animals vs controls.^75^ However, vaccines designed to induce T-cell responses have not uniformly increased HCV control; data indicate that not all vaccine-induced immune responses are beneficial. Vaccination of chimpanzees with recombinant NS3, NS4, and NS5 proteins formulated with the ISCOMATRIX adjuvant resulted in persistent HCV infection on rechallenge in all 5 vaccinated animals, despite the appearance of HCV-specific CD4^+^ and CD8^+^ T cells in liver before challenge and infection.^76^ When naïve chimpanzees were immunized with DNA plasmids expressing the core-E1-E2 and NS3 for priming, and with recombinant modified vaccinia (MVA)-expressing core-E1-E2 and NS3 gene sequences as a boost, they developed HCV-specific antibody and T-cell responses.^77^ Despite these immune responses, vaccinated animals had a higher rate of virus persistence than control animals.

The observation that vaccines have failed even when they induced T-cell responses indicates that trials should proceed with caution and should include strategies to identify the features of immune responses associated with control. In general, the small number of chimpanzees tested, the diversity of the vaccines tested, and the different methods used to assess induced immune responses make it exceedingly difficult to determine what aspects of the elicited immune responses provided protection. It is also unclear if the results from nonhuman primate studies can be translated to humans. More detailed phenotypic and functional analyses, performed on existing and future trial specimens, are required to identify factors that determine whether a vaccine will reduce the rate of persistent infection in humans.

Two vaccines designed to prevent infection solely by eliciting T-cell–mediated immunity have been tested in phase I (safety and immunogenicity) trials in human volunteers not at risk for HCV infection. A prototype vaccine with the HCV core protein and ISCOMATRIX adjuvant was assessed for its ability to induce T-cell responses in healthy individuals not at risk for HCV infection.^78^ Although the vaccine was generally well tolerated, CD8^+^ T-cell responses were detected in only 2 of the 8 participants receiving the highest dose. The second vaccine tested in healthy volunteers and designed to elicit T-cell responses is the foundation of the only candidate vaccine to have advanced to a trial in at-risk subjects. This vaccine is composed of a replication-defective chimpanzee adenovirus (ChAd) vector encoding NS3, NS4, and NS5 proteins.^79^

Replication-defective adenovirus vectors have been used to introduce antigens from other pathogens and induce a potent immune response. Human adenovirus serotype 5 (Ad5) induces protective immune responses against diverse pathogens and cancer in animal models and elicits robust and sustained cellular immunity in humans. However, most humans have neutralizing antibodies to Ad5, which can reduce immune responses to the antigens carried in Ad5-based vaccines. Replication-defective adenoviral vectors based on serotype 6 (subgroup C) and 24 (subgroup D) have low seroprevalence in humans, reducing immunologic cross-reactivity.^66^ A segment of DNA coding for NS3–5 of the HCV genotype 1b was delivered by Ad6 followed by Ad24 and finally by electroporated plasmid DNA in chimpanzees.^73^ Following HCV rechallenge with HCV genotype 1a virus, all vaccinated chimpanzees had a significantly reduced peak of viremia, with the average peak more than 100-fold lower in the HCV vaccination group than in the control group.

These viral kinetics are similar to those observed in PWID who successfully control HCV with repeated exposure.^40^ All 5 animals that received the vaccine had minimal increases in levels of alanine amino transferase compared with mock-vaccinated control chimpanzees challenged with HCV. Four of 5 vaccinated chimpanzees cleared the virus with a significantly shorter duration of viremia vs the control group, whereas 1 vaccinated chimpanzee maintained low levels of HCV RNA for the duration of the study. A follow-up study revealed that after challenge, vaccinated chimpanzees had early expansion of CD8^+^ T cells with higher expression of the memory precursor molecule CD127, lower levels of the inhibitory molecule PD1, and increased effector functions compared with primary T cells from the mock-vaccinated controls that developed persistent infections.^80^ Early expansion of CD127^+^ HCV-specific T cells with high functionality was previously demonstrated in chimpanzees that spontaneously controlled acute HCV infection.^81^

Based on these promising results, replication-defective vectors generated from a subset of novel ChAd serotypes were screened to determine whether they were neutralized by human sera and if they were able to grow in human cell lines.^66^ Of these, ChAd3 expressing the NS region from HCV was found to induce long-lasting T- and B-cell memory responses in mice and macaques. Adenoviral vectors expressing NS proteins from HCV genotype 1b were constructed based on the rare serotypes Ad6 and ChAd3. The Ad6-NS and ChAd3-NS vaccines were tested in an adenovirus prime heterologous adenovirus boost regimens in a safety and immunogenicity phase 1 clinical trial in healthy volunteers not at risk for HCV infection.^82^ ChAd3-NS was well tolerated and highly immunogenic, with intracellular cytokine staining demonstrating that ChAd3-NSmut primed a large number of polyfunctional CD8^+^ T cells. Antigen-specific polyfunctional CD4^+^ T cells were detected at a lower frequency. Memory CD8^+^ T cells that expressed CD127, but not PD1, were sustained in circulation. Although more robust recognition of HCV genotype 1b peptides (matching the vaccine) was observed, there was recognition of genotype 1a and 3a peptide pools that might provide cross-genotypic protection. Responses were sustained for at least 1 year after boosting with the heterologous adenoviral vector, demonstrated by the persistence of central and effector memory T-cell pools that retained polyfunctionality and proliferative capacity. This response was similar to the sustained T-cell responses (in magnitude and quantity) associated with protective immunity in vaccine trials of other pathogens. However, boosting was not as robust with heterologous adenovirus as it was later found to be with MVA, so the vaccine was advanced to HCV at-risk subjects with ChAd3 prime and MVA boost, both expressing NS3–5.^79^

The ChAd3-NS prime and MVA-NS boost strategy is being evaluated in a staged phase 1/2 study, under way in Baltimore, San Francisco, and New Mexico (see ClinicalTrials.gov NCT01436357). Vaccine responses were primed with the ChAd3-NS at a dose previously found to be well tolerated and immunogenic in healthy volunteers, followed by boosting 8 weeks later with MVA-NS. The primary endpoint of this study is to prevent HCV persistence in HCV-naïve populations of PWID at high risk for infection. Phase 1 of the trial was completed with safety signal and immunogenicity parameters supporting advancement, and enrollment in phase 2 of the trial began in 2013. Results are expected in 2019. If promising, confirmation that the vaccine reduces the rate of chronic HCV infection in PWID would likely require larger trials; however, this trial will at minimum demonstrate the feasibility of conducting HCV vaccine trials in PWID.

## Neutralizing Antibody-mediated Protection

Many licensed prophylactic vaccines against other viral infections induce neutralizing or binding antibody titers that correlate with protection. Although HCV envelope genes are extremely diverse, there is evidence from infected humans and animal models that neutralizing antibodies can be protective. Clearance of HCV infection is associated with the early development of serum antibodies capable of blocking infection by multiple heterologous HCV strains. Antibodies with these characteristics are called broadly neutralizing antibodies (bNAbs).^83^, ^84^, ^85^ The appearance of NAbs later in infection also has been associated with spontaneous clearance of HCV infection.^86^ Individuals who clear their first infection clear subsequent infections more than 80% of the time. Along with expanded T-cell responses, clearance of reinfection was associated with rapid induction of antibodies capable of neutralizing heterologous HCV strains.^40^ bNAbs have been isolated from the B cells of individuals who cleared HCV infection without treatment.^87^, ^88^ Some of these bNAbs have low levels of somatic hypermutation, similar to levels commonly stimulated by vaccination against other viruses, so vaccine induction of bNAbs against HCV might be feasible.^89^, ^90^

There is also evidence of antibody protection against HCV challenge in animal models of infection. Infusion of immunoglobulin isolated from the serum of a chronically infected human before challenge with homologous virus from the same donor prevented infection of most human liver chimeric mice. Similarly, infusion of chronic-phase human immunoglobulin before challenge of a chimpanzee prevented infection with homologous, but not heterologous HCV strains.^91^, ^92^, ^93^ In contrast, it was demonstrated that infusion of bNAbs before challenge with heterologous virus could partially or fully prevent infection in humanized mice^94^, ^95^, ^96^ and chimpanzees,^97^ and combinations of bNAbs also abrogated established HCV infection in a human liver chimeric mice.^98^ Together, these studies demonstrate that typical chronic-phase human sera have protective titers of strain-specific NAbs, but bNAbs may be necessary to prevent infection by diverse, heterologous HCV strains.

## Anti-HCV bNAb Epitopes and Implications for Vaccine Design

The targets of the NAb response against HCV are the viral envelope glycoproteins, E1 and E2. E1 and E2 are membrane-anchored proteins that are believed to form a heterodimer on the surface of viral particles. Although the function of E1 is unclear, E2 interacts with multiple cell-surface receptors, including but not limited to CD81 and scavenger receptor class B member 1, which mediates viral entry.^99^ Hypervariable region 1 (HVR1), a 27-amino acid region at the N-terminus of E2, is an immunodominant epitope that evolves rapidly under antibody pressure.^100^, ^101^ Although most HCV-infected individuals develop strain-specific NAbs against HVR1, viral mutations generally confer resistance to these antibodies.^18^, ^102^, ^103^, ^104^, ^105^, ^106^

**Figure 2:**
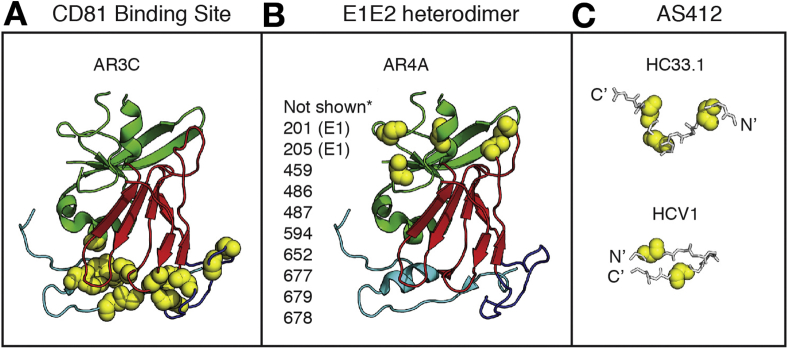
bNAb epitopes are conformationally complex and flexible. (*A*, *B*) The crystallized structure of a truncated HCV E2 protein, strain H77, PDB 4MWF, from Kong et al,^119^ with E2 domains and bNAb binding residues highlighted in Pymol, v1.8.6.2. The E2 front layer is *cyan*, central beta sheet is *red*, and the back layer is *green*. Binding residues of the CD81 binding site-targeting bNAb AR3C (*A*) or the E1E2 complex-targeting bNAb AR4A (*B*), identified by alanine scanning mutagenesis and binding assays, are marked with *yellow spheres*.^116^ Amino acid side chains are not shown. *Putative AR4A binding residues not present in the E2 crystal structure are listed next to the structure. Binding residues shown or listed are positions at which mutation to alanine had the greatest effect on AR3C or AR4A binding, but minimal effect on control mAbs. Numbering is relative to the H77 polyprotein sequence. (*C*) Structures of peptide epitopes crystallized in complex with AS412-targeting bNAbs HC33.1 or HCV1. Fab structures are not shown. HC33.1 binds the peptide in an extended conformation (PDB 4XVJ),^127^ whereas HCV1 binds the peptide in a hairpin conformation (PDB 4DGY).^113^ Critical binding residues for each bNAb determined by alanine scanning mutagenesis and binding assays are marked with *yellow spheres*.^110^, ^120^ Amino acid side chains are not shown.

Unlike strain-specific NAbs that target HVR1, bNAbs target relatively conserved epitopes on E2 or on the E1E2 heterodimer.^87^, ^94^, ^96^, ^107^, ^108^, ^109^, ^110^, ^111^, ^112^, ^113^, ^114^ Characterization of these bNAbs has helped to identify epitopes that could be incorporated into a vaccine. These studies have also identified challenges that may limit induction of bNAbs, including complex conformational bNAb epitopes and structural flexibility of envelope proteins. Pierce et al^115^ and Gopal et al^116^ performed comprehensive alanine scanning mutagenesis across the entire E2 or E1E2 genes followed by binding assays with many bNAbs. These studies identified the binding epitopes of many bNAbs and non-neutralizing antibodies (reviewed in Kinchen et al^117^ and Fuerst et al^118^), demonstrating that antibodies that interact with the region of E2 commonly referred to as the back layer are generally weakly neutralizing or non-neutralizing. In contrast, most bNAbs do not interact with the E2 back layer, but instead bind at the CD81 receptor binding site of E2 (CD81bs) or to the E1E2 heterodimer (Figure 2).

The x-ray crystal structure of a truncated strain H77 E2 core, complexed with a CD81bs bNAb designated AR3C, provided the first view of a bNAb in complex with a near full-length, correctly folded envelope protein.^119^ This structure, along with the previously described binding studies, demonstrated that the AR3C conformational epitope is structurally complex, with extensive AR3C binding residues in multiple E2 domains, including the front layer and the CD81 binding loop (Figure 2*A*). BNAbs targeting the E1E2 heterodimer, such as the bNAb designated AR4A, recognize epitopes that are still ill-defined, but they also appear to bind to residues spanning multiple envelope protein domains (Figure 2*B*).^96^, ^98^ A third group of bNAbs targets continuous epitopes spanning amino acids 412 to 423, a region commonly referred to as AS412, near the CD81bs of E2 (Figure 2*C*).^110^, ^120^, ^121^, ^122^, ^123^ Crystal structures of peptides spanning amino acids 412 to 423 in complex with monoclonal antibodies (mAbs) HCV-1, HC33.1, AP33, and 3/11 demonstrate that mAbs can bind to this region with different angles of approach.^113^, ^124^, ^125^, ^126^, ^127^ In addition, the region assumes an extended conformation in some complexes and a beta hairpin conformation in others, indicating that it is structurally flexible. It is not clear whether any of these epitope conformations predominate in vivo. Overall, these characterizations of bNAbs help to define epitopes that could be incorporated into a vaccine, but they also identify the challenge of discontinuous, conformational epitopes that might be difficult to reproduce for a vaccine, and structural flexibility of some epitopes that could limit bNAb induction.

## Trials of Vaccines Designed to Induce a Humoral Response

Strategies developed to induce antibodies against HCV have included protein-based, DNA-based, VLP-based, pox virus–based, and whole virus–based vaccines. A vaccine composed of recombinant full-length E1E2 protein from a single genotype 1a HCV strain with oil-in-water adjuvant has been tested in rodents, nonhuman primates, and humans. This vaccine induced fairly strong heterologous neutralizing activity in guinea pigs,^128^ and protected chimpanzees against homologous HCV challenge.^129^ Although the vaccine did not provide sterilizing immunity, it also reduced rates of persistence in chimpanzees after challenge with a neutralization sensitive heterologous virus.^76^ However, post-vaccination bNAb titers were detectable in only 3 of 16 vaccinees in a phase 1a human trial despite induction of strong CD4^+^ T-cell proliferation in response to recombinant E1E2. T vaccine antigen or adjuvant might therefore require optimization.^130^, ^131^

Studies of other vaccines in chimpanzees, such as DNA vaccines designed to express envelope proteins, DNA priming followed by MVA boost, or expression of envelope proteins on VLPs, have induced relatively disappointing humoral responses (reviewed in Liang^132^ and Bukh^15^). Two chimpanzees given a DNA vaccine designed to express cell-surface E2 protein developed low levels of E2-specific antibodies, but it is not clear that the low titers of induced antibodies had a significant role in clearance.^133^ The DNA priming followed by MVA boost was designed to express core, E1, E2, and NS3 proteins, but failed to induce detectable NAb titers.^77^ The VLPs (HCV core, E1, and E2 proteins) did not induce an antibody response, or produced barely detectable antibodies against core and E1E2, although the chimpanzees developed robust T-cell responses against these proteins.^62^ In contrast, this same vaccine induced production of envelope-specific antibodies in baboons, although neutralizing titers were not assessed.^67^ There are several reasons that chimpanzees could have weak NAb responses; for example, HCV-infected chimpanzees generally mount less vigorous anti-envelope antibody responses than infected humans.^134^, ^135^, ^136^

As the efficiency of production of chimeric replication competent cell culture viruses (HCVcc) has improved, use of whole inactivated virus has also become a more viable vaccine strategy, although there are limitations to this strategy. Vaccination of mice with inactivated J6/JFH-1 HCVcc stimulated NAbs against homologous virus and 2 heterologous HCV strains, and infusion of purified immunoglobulin from vaccinated mice prevented homologous virus infection of mice with humanized livers.^137^ Other groups have developed multivalent VLP or purified recombinant E1E2 protein vaccines that express envelope genes from multiple genotypes. In early studies, both vaccines induced cross-reactive antibodies in mice, and the multivalent E1E2 protein vaccine induced NAbs against 1 homologous and 1 heterologous HCV strain.^63^, ^138^ Further development of vaccines expressing full-length, native E1E2 is supported by a recent study demonstrating that combinations of human NAbs against multiple epitopes can display complementary neutralizing breadth and in some cases neutralizing synergy.^139^

Truncated protein antigens and rationally designed peptide antigens are also at early stages of development. Studies showing that HVR1 may occlude conserved bNAb epitopes^140^ led Vietheer et al^141^ to develop an E2 vaccine antigen with deletion of 3 variable regions. This vaccine induced moderately high titers of bNAbs in guinea pigs.^141^ However, Law et al^142^ found that full-length E1E2 or a variant with truncated HVR1 induced equivalent titers of NAbs against heterologous HCV strains in vaccinated guinea pigs, so truncation of HVR1 may not be necessary. Recently, Pierce et al^143^ developed a molecular scaffold to present the AS412 epitope. Antibody responses in vaccinated mice were modest, so further optimization may be needed to better match the structure of this bNAb epitope. Overall, more work is needed to identify ideal vaccine antigens, as well as optimal vaccine adjuvants. It will be important to perform many trials, evaluating as many vaccine approaches as possible; it is unclear whether anti-HCV antibody responses observed in rodents or nonhuman primates will also occur in humans.

## HCV Panels Used to Quantify Neutralizing Breadth

Accurate measurement of neutralizing breadth of sera is critical to assess antibody responses induced by candidate vaccines. An ongoing challenge that significantly limits comparison of results among preclinical vaccine studies is the use of relatively few HCV strains to assess neutralizing breadth of antibodies, as well as the use of different HCV strains and different neutralization assays in each laboratory.^96^, ^107^, ^144^, ^145^, ^146^, ^147^ Studies have demonstrated that neutralization sensitivity varies widely among HCV strains, within and across HCV genotypes. In a study by Carlsen et al^25^ 50% inhibitory concentrations of the same mAb among different HCVcc strains varied more than 1000-fold, within and among HCV genotypes. Urbanowicz et al^148^ measured neutralization of more than 70 genotype 1-6 HCV pseudoparticles (HCVpp), which are retroviral particles with HCV envelope proteins on their surface, by 5 mAbs. They also observed wide variation in neutralization sensitivity, within and among subtypes. These findings indicate that the neutralization sensitivity of individual strains from any genotype are not representative of the genotype as a whole. Therefore, it is critical that neutralization panels include as many diverse HCV strains as possible. Moreover, accurate comparison of candidate vaccines would be greatly facilitated by testing of all sera against a more standardized panel of heterologous HCV strains.

## Future Directions

There are significant deficits in our tools for development of an HCV vaccine. Elucidating the mechanisms through which antigen-specific immune cell populations mediate long-term protection is an important goal. Vaccine strategies meant to overcome the enormous diversity of HCV must generate a broad immune response, capable of responding to abundant variations. Selecting antigens to maximize the induction of T-cell and antibody responses that elicit successful responses remains an active area of research. At least 4 types of HCV antigens have been proposed to maximize induction of robust and cross-protective responses: specific previously defined T-cell epitopes for inclusion, a single circulating HCV variant quadrivalent genotype 1a/1b/2a/3a VLP, and antigens generated computationally that minimize the degree of sequence dissimilarity between a vaccine strain and contemporary circulating viruses.

One study demonstrated the potential of computer-generated sequences to elicit cross-reactive T-cell responses, compared with previously defined T-cell epitopes or circulating HCV variants. These data support the use of a synthetically generated sequence as vaccine antigen to elicit robust CD8^+^ T-cell responses.^149^ However, computational strategies to aid in antigen design have not been used in HCV vaccines. Methods for introducing antigens are also being explored, with novel vectors in development for use against HCV and other viral targets. Studies in small animal models and macaques have shown that the addition of class-II invariant chain to an immunogen encoded in adenoviral and MVA viral vectors may significantly increase specific responses of CD4^+^ and CD8^+^ T cells to HCV. Phase 1 studies are under way to assess this strategy in healthy volunteers, with results expected in 2019. EudraCT Number: 2016-000983-41 (Capone et al^150^).

Going forward, successful control of HCV infection will most likely require a combination of large-scale screening to identify infected individuals, treatment of infected persons, and prevention and harm-reduction strategies for those who are uninfected and at risk. Although pharmaceutical companies have invested more in in drug development, vaccine development requires investment from sources beyond government and charitable foundations. A prophylactic HCV vaccine is an important part of a successful strategy for global control. Although development is not easy, the quest is a worthy challenge.
